# The impact of old age on cancer-specific and non-cancer-related survival following elective potentially curative surgery for Dukes A/B colorectal cancer

**DOI:** 10.1038/sj.bjc.6604669

**Published:** 2008-09-16

**Authors:** D C McMillan, D J Hole, C S McArdle

**Affiliations:** 1 University Department of Surgery, Faculty of Medicine, University of Glasgow, Glasgow G12 8QQ, UK; 2 University Department of Public Health, Faculty of Medicine, University of Glasgow, Glasgow G12 8QQ, UK

**Keywords:** colorectal cancer, age, elective, curative surgery, survival

## Abstract

Previous studies have suggested that survival following surgery for colorectal cancer is poorer in the elderly. However, the findings were inconsistent and none of the studies adjusted for case mix. The aim of this study was to establish whether there were age-related differences in cancer (colorectal)-specific and non-cancer (colorectal)-related survival in patients undergoing elective potentially curative resection for Dukes stage A/B colorectal cancer. One thousand and forty three patients who underwent elective potentially curative resection for Dukes’ A/B colorectal cancer between 1991 and 1994 in 11 hospitals in Scotland were included in the study. Ten year cancer-specific and non-cancer-related survival and the hazard ratios were calculated according to age groups (<64; 65–74/>74 years). On follow-up 273 patients died of their cancer and 328 died of non-cancer-related causes. At 10 years, overall survival was 45%, cancer specific was 70% and non-cancer-related survival was 64%. On multivariate analysis of all factors, age (HR 1.38, 95% CI 1.18–1.62, *P*<0.001), sex (HR 1.74, 95% CI 1.36–2.23, *P*<0.001), site (HR 1.42, 95% CI 1.11–1.81, *P*<0.01) and Dukes’ stage (HR 1.71, 1.19–2.47, *P*<0.01) were independently associated with cancer-specific survival. On multivariate analysis of all factors, age (HR 2.14, 1.84–2.49, *P*<0.001), sex (HR 1.43, 1.15–1.79, *P*<0.01) and deprivation (HR 1.30, 1.09–1.55, *P*<0.01) were independently associated with non-cancer-related survival. The results of this study show that increasing age impacts negatively both on cancer-specific and non-cancer-related survival following elective potentially curative resection for node-negative colorectal cancer. However, the effect of increasing age is greater on the non-cancer-related survival. These results suggest that cancer-specific and non-cancer-related mortality should be considered separately in survival analysis of these cancer patients.

Colorectal cancer is the second commonest cause of cancer death in Western Europe and North America. Many patients have evidence of locally advanced or metastatic disease at the time of initial presentation. Even in those undergoing apparently curative resection, only half survive for 5 years (McArdle and Hole, 2002a).

It has long been recognised that there are a number of factors, in addition to pathological stage, which contribute to poor outcome following potentially curative surgery for colorectal cancer. Age (Mulcahy *et al*, 1994; Shankar and Taylor, 1998; Colorectal Cancer Collaborative Group, 2000), gender (McArdle *et al*, 2003), deprivation (Hole and McArdle, 2002), tumour site (McArdle and Hole, 2002b), emergency presentation (McArdle and Hole, 2004a) and specialisation (McArdle and Hole, 2004b) have been shown to impact on long-term survival in these patients.

However, whether old age is associated with poorer survival, independent of these factors, remains unclear. The aim of this study was to establish whether there were age-related differences in cancer (colorectal)-specific and non-cancer (colorectal)-related survival in patients undergoing elective potentially curative resection for Dukes stage A/B colorectal cancer.

## Patients and methods

One thousand and forty three patients who underwent an elective potentially curative resection for Dukes A/B colorectal cancer between 1 January 1991 and 31 December 1994 in 11 hospitals in the central belt of Scotland were included in the study. Information was abstracted from casenotes by two specially trained data managers. Details included age, sex, deprivation category, site of tumour, extent of tumour spread, the nature of surgery, post-operative mortality, Dukes’ stage and adjuvant therapy. Data for 1991 and 1992 were collected retrospectively, and those for 1993 and 1994 were collected prospectively. There was no difference in baseline characteristics of the patients between the two periods.

The extent of deprivation was defined using the Carstairs Index (Carstairs and Morris, 1991), an area-based measure derived from the 1991 census data based on the postcode of residents at diagnosis. Carstairs divides the scores into a seven-point scale ranging from most affluent (category 1) to most deprived (category 7).

Tumours were classified according to the site colon or rectum. The extent of tumour spread was assessed by conventional Dukes’ classification based on histological examination of the resected specimen.

Patients were deemed to have had a curative resection if the surgeon considered that there was no macroscopic residual tumour once resection had been completed.

Individual surgeons were defined as specialists or non-specialists by a panel of six senior consultants and one of the authors (CSMcA). Two of the six consultants were specialist colorectal surgeons from teaching hospitals and four were district general hospital consultants. These assessments were made without the knowledge of the outcome and before any analysis was performed.

The approval was obtained for information on date and cause of death to be checked with that received by the cancer registration system through linkage with the Registrar General (Scotland). Deaths up to the end of 2003 have been included in the analysis, providing an average length of follow-up of 11 years (minimum 9 years, maximum 13 years).

### Statistical analysis

The percentages of patients surviving 10 years were calculated using the Kaplan–Meier technique. Comparison of the association between age and other variables was made using the *χ*^2^ test or a *χ*^2^ for trend where appropriate. The effect of age on cancer- and non-cancer-related survival was examined using Cox's proportional hazards model. Analysis was performed using the SPSS software package (SPSS Inc., Chicago, IL, USA).

## Results

Of the 1043 patients included in the analysis, 33% were aged 75 years or over, 21% were socioeconomically deprived, 59% had colonic tumours and 82% had Dukes’ B disease at the time of surgery. Two hundred and seventy five (26%) patients were treated by a specialist surgeon. Three percent of patients received adjuvant therapy.

The baseline characteristics of the patients included in the study are shown in Table 1. With increasing age there was an increase in the proportion of patients who had colonic tumours (*P*<0.001). Few patients over the age of 74 years received adjuvant therapy (*P*<0.001).

Two hundred and seventy three patients died of their cancer and 328 died of non-cancer-related causes (Table 2). Of the non-cancer-related deaths, 212 (65%) patients died of cardiovascular or respiratory disease. At 10 years, overall survival was 45%, cancer specific was 70% and non-cancer-related survival was 64%. An increased proportion of cancer deaths at 10 years was associated with increased Dukes’ stage (*P*<0.05) and non-specialist surgeons (*P*<0.10). An increased proportion of non-cancer-related deaths at 10 years was associated with older age (*P*<0.001), female gender (*P*<0.05) and deprivation (*P*<0.10).

On univariate analysis, age (*P*<0.01), sex (*P*<0.001), site (*P*<0.05) and Dukes’ stage (*P*<0.01) were significantly associated with cancer-specific survival (Table 3). On multivariate analysis of all factors, age (HR 1.38, 95% CI 1.18–1.62, *P*<0.001), sex (HR 1.74, 95% CI 1.36–2.23, *P*<0.001), site (HR 1.42, 95% CI 1.11–1.81, *P*<0.01) and Dukes’ stage (HR 1.71, 1.19–2.47, *P*<0.01) were independently associated with cancer-specific survival.

On univariate analysis, age (*P*<0.001), sex (*P*<0.05), deprivation (*P*<0.01) and adjuvant therapy (*P*<0.10) were significantly associated with non-cancer-related survival (Table 4). On multivariate analysis of all factors, age (HR 2.14, 1.84–2.49, *P*<0.001), sex (HR 1.43, 1.15–1.79, *P*<0.01) and deprivation (HR 1.30, 1.09–1.55, *P*<0.01) were independently associated with non-cancer-related survival.

The relationship between age and the hazard ratios for cancer and non-cancer-related survival in patients with Dukes’ A/B disease colon and rectum cancer is shown in Table 5. Compared with patients under the age of 64 years who had colonic tumours, those patients over the age of 74 years had a hazard ratio of 2.2 (*P*<0.01) and 5.4 (*P*<0.001) for cancer and non-cancer-related survival, respectively. Compared with patients under the age of 64 years who had rectal tumours, those patients over the age of 74 years had a hazard ratio of 5.1 (*P*<0.001) for non-cancer-related survival.

## Discussion

The results of this study show that, in patients undergoing elective potentially curative surgery for Dukes’ A/B colorectal cancer, with 10-year follow-up, more patients die of intercurrent disease, in particular cardiovascular or respiratory disease, than die of cancer. Increasing age, 75 years and over, impacted on cancer-related and non-cancer-related survival. Although, it was notable that the effect on non-cancer-related survival was approximately twice that of cancer-related survival.

Given that colorectal cancer is a common cancer and that large numbers of patients undergoing elective potentially curative resection will die of intercurrent comorbid disease, the use of overall survival as an end point may give misleading results. Therefore, future studies on these patients should specify the associations between factors and cancer-specific survival and non-cancer-related survival separately to establish whether the factor impacts equally on cancer-specific and non-cancer-related survival.

The results of this study have a number of important implications. First, with the introduction of screening programmes for colorectal cancer and the consequent increase in early stage disease, it is likely that the issue of age-related comorbid death will become a major issue in these patients. If this were proven to be the case, it would be important that within screening programmes for colorectal cancer, patients should also be screened for the presence of comorbid disease. For example, in patients aged of 75 years and over undergoing potentially curative resection for node-negative colorectal cancer, it would be important not only to treat the cancer, but also the comorbid disease, in particular it would appear from this study that this is cardiovascular or respiratory disease.

In the context of this study, it is of interest that a strong association between colorectal cancer and vascular disease has been reported (Chan *et al*, 2006, 2007). This may be because of the shared environmental risk factors such as age, smoking, diabetes mellitus and obesity (Giovannucci and Martínez, 1996; Larsson *et al*, 2005; McMillan *et al*, 2006). It has also been hypothesised that these diseases develop through a common pathway of chronic inflammatory disease (Chan *et al*, 2006, 2007). Indeed, there is evidence that such environmental factors are directly associated with C-reactive protein concentrations, the prototypical marker of the systemic inflammatory response, in apparently healthy subjects (Koenig *et al*, 1999; Hutchinson *et al*, 2000; Chenillot *et al*, 2000: Wong *et al*, 2001). However, it remains to be determined whether the systemic inflammatory response impacts equally on cancer-specific and non-cancer-related survival in patients undergoing potentially curative surgery for colorectal cancer.

In summary, the results of this study show that although age impacts both on cancer-specific and non-cancer-related survival following elective potentially curative resection for node-negative colorectal cancer, the effect is greater on non-cancer-related survival. These results suggest that cancer-specific and non-cancer-related deaths should be considered separately in survival analysis of these patients.

## Figures and Tables

**Table 1:** Baseline characteristics of patients undergoing elective curative resection for Dukes A/B colorectal cancer by age (*n*=1043)

	**Age (years)**			
	**<64 years *n*=305 (29%)**	**65–74 years *n*=398 (38%)**	**>74 years *n*=340 (33%)**	***P*-value**
Female	143 (47%)	186 (47%)	184 (56%)	
Male	162 (53%)	212 (53%)	156 (46%)	0.061
				
*Depcat score*				
1, 2	53 (17%)	70 (18%)	69 (20%)	
3, 4, 5	191 (63%)	245 (62%)	201 (59%)	
6, 7	61 (20%)	83 (21%)	70 (21%)	0.625
				
*Site*				
Colon	160 (52%)	228 (57%)	223 (66%)	
Rectum	145 (48%)	170 (43%)	117 (37%)	<0.001
				
*Dukes’ stage*				
A	52 (17%)	76 (19%)	56 (17%)	
B	253 (83%)	322 (81%)	284 (83%)	0.822
				
*Specialisation*				
Specialist	76 (25%)	104 (27%)	95 (28%)	
Non-specialist	223 (75%)	288 (73%)	240 (72%)	0.402
				
*Adjuvant therapy*				
Yes	15 (5%)	16 (4%)	3 (1%)	
No	290 (95%)	382 (96%)	337 (99%)	<0.001

**Table 2:** Baseline characteristics of patients undergoing elective curative resection for Dukes A/B colorectal cancer-by-cancer and non-cancer death after 10 years follow-up

	**Cancer deaths (*n*=273)**	**Non-cancer deaths (*n*=328)**	***P*-value**
Age			
<64 years	68 (25%)	42 (13%)	
65–74 years	109 (40%)	129 (39%)	
>74 years	96 (35%)	157 (48%)	<0.001
			
Female	104 (38%)	153 (47%)	
Male	169 (62%)	175 (53%)	0.035
			
*Depcat score*			
1, 2	53 (19%)	45 (14%)	
3, 4, 5	167 (62%)	206 (63%)	
6, 7	53 (19%)	77 (24%)	0.052
			
*Site*			
Colon	148 (54%)	192 (59%)	
Rectum	125 (46%)	136 (41%)	0.287
			
*Dukes’ stage*			
A	34 (13%)	61 (19%)	
B	239 (87%)	267 (81%)	0.040
			
*Specialisation*			
Specialist	61 (23%)	94 (27%)	
Non-specialist	209 (77%)	226 (71%)	0.062
			
*Adjuvant therapy*			
Yes	10 (4%)	6 (2%)	
No	263 (96%)	322 (98%)	0.165

**Table 3:** The relationship between clinicopathological characteristics and cancer-specific survival in patients undergoing elective curative resection for Dukes A/B colorectal cancer: univariate and multivariate analysis

		**Univariate analysis**		**Multivariate analysis**	
	**Patients (*n*=1043)**	**Hazard ratio (95% CI)**	***P*-value**	**Hazard ratio (95% CI)**	***P*-value**
Age (<64/65–74/>74 years)	305/398/340	1.29 (1.11–1.50)	0.001	1.38 (1.18–1.62)	<0.001
Sex (female/male)	513/530	1.75 (1.37–2.23)	<0.001	1.74 (1.36–2.23)	<0.001
Deprivation (1–2/3–5/6–7)^a^	192/637/214	0.98 (0.81–1.19)	0.840		0.322
Site (colon/rectum)	611/432	1.28 (1.01–1.62)	0.044	1.42 (1.11–1.81)	0.006
Dukes’ stage (A/B)	184/859	1.70 (1.19–2.44)	0.004	1.71 (1.19–2.47)	0.004
Specialisation (yes/no)	275/751	1.27 (0.95–1.69)	0.101		0.104
Adjuvant therapy (yes/no)	34/1009	0.94 (0.50–1.77)	0.845		0.750

a Individual deprivation categories were used in the statistical analysis.

**Table 4:** The relationship between clinicopathological characteristics and non-cancer survival in patients undergoing elective curative resection for Dukes A/B colorectal cancer: univariate and multivariate analysis

		**Univariate analysis**		**Multivariate analysis**	
	**Patients (*n*=1043)**	**Hazard ratio (95% CI)**	***P*-value**	**Hazard ratio (95% CI)**	***P*-value**
Age (<64/65–74/>74years)	305/398/340	2.11 (1.82–2.45)	<0.001	2.14 (1.84–2.49)	<0.001
Sex (female/male)	513/530	1.32 (1.06–1.64)	0.012	1.43 (1.15–1.79)	0.002
Deprivation (1–2/3–5/6–7)^a^	192/637/214	1.30 (1.09–1.53)	0.003	1.30 (1.09–1.55)	0.003
Site (colon/rectum)	611/432	1.11 (0.88–1.37)	0.407		0.094
Dukes’ stage (A/B)	184/859	1.07 (0.81–1.42)	0.676		0.346
Specialisation (yes/no)	275/751	0.89 (0.70–1.13)	0.332		0.548
Adjuvant therapy (yes/no)	34/1009	2.11 (0.96–4.87)	0.061		0.302

a Individual deprivation categories were used in the statistical analysis.

**Table 5:** Hazard ratios for cancer-specific and non-cancer survival at 10 years in patients undergoing elective curative resection for Dukes A/B colorectal cancer by age

	**Cancer specific (deaths=148)**		**Non-cancer (deaths=192)**	
**Colon (*n*=611)**	**Hazard ratio (95% CI)**	***P*-value**	**Hazard ratio (95% CI)**	***P*-value**
*Age*				
<64 years	1	0.004	1	<0.001
65–74 years	1.71 (1.09–2.69)	0.019	3.01 (1.85–4.91)	<0.001
>74 years	2.16 (1.38–3.37)	0.001	5.39 (3.36–8.64)	<0.001
				
	**Cancer specific (deaths=125)**		**Non-cancer (deaths=136)**	
**Rectum (*n*=432)**	**Hazard ratio (95%CI)**	***P*-value**	**Hazard ratio (95%CI)**	***P*-value**
*Age*				
<64 years	1	0.286		<0.001
65–74 years	1.28 (0.85–1.94)	0.237	3.02 (1.84–4.50)	<0.001
>74years	1.42 (0.90–2.25)	0.132	5.09 (3.08–8.41)	<0.001
